# Solid Lipid Nanoparticles: A Potential Multifunctional Approach towards Rheumatoid Arthritis Theranostics

**DOI:** 10.3390/molecules200611103

**Published:** 2015-06-16

**Authors:** João Albuquerque, Catarina Costa Moura, Bruno Sarmento, Salette Reis

**Affiliations:** 1 UCIBIO, ICETA REQUIMTE, Faculty of Pharmacy, University of Porto, Rua de Jorge Viterbo Ferreira, 228, 4050-313 Porto, Portugal; E-Mails: joao.albuquerque.costa@gmail.com (J.A.); cmoura@ff.up.pt (C.C.M.); shreis@ff.up.pt (S.R.); 2 Biocarrier Group, INEB—Instituto de Engenharia Biomédica, Rua do Campo Alegre, 823, 4150-180 Porto, Portugal; 3 CESPU, Instituto de Investigação e Formação Avançada em Ciências e Tecnologias da Saúde, Rua Central de Gandra, 1317, 4585-116 Granda PRD, Portugal

**Keywords:** nanomedicine, rheumatoid arthritis, theranostics, solid lipid nanoparticles

## Abstract

Rheumatoid arthritis (RA) is the most common joint-related autoimmune disease and one of the most severe. Despite intensive investigation, the RA inflammatory process remains largely unknown and finding effective and long lasting therapies that specifically target RA is a challenging task. This study proposes a different approach for RA therapy, taking advantage of the new emerging field of nanomedicine to develop a targeted theranostic system for intravenous administration, using solid lipid nanoparticles (SLN), a biocompatible and biodegradable colloidal delivery system, surface-functionalized with an anti-CD64 antibody that specifically targets macrophages in RA. Methotrexate (MTX) and superparamagnetic iron oxide nanoparticles (SPIONs) were co-encapsulated inside the SLNs to be used as therapeutic and imaging agents, respectively. All the formulations presented sizes under 250 nm and zeta potential values lower than −16 mV, suitable characteristics for intravenous administration. Transmission electron microscopy (TEM) photographs indicated that the SPIONs were encapsulated inside the SLN matrix and MTX association efficiency values were higher than 98%. *In vitro* studies, using THP-1 cells, demonstrated that all formulations presented low cytotoxicity at concentrations lower than 500 μg/mL. It was proven that the proposed NPs were not cytotoxic, that both a therapeutic and imaging agent could be co-encapsulated and that the SLN could be functionalized for a potential future application such as anti-body specific targeting. The proposed formulations are, therefore, promising candidates for future theranostic applications.

## 1. Introduction

Rheumatoid arthritis (RA) is a chronic systemic inflammatory disease of uncertain cause that affects 1% of the population of the developed world, being more common among women than men, with 80% of the total cases occurring between the ages of 35 and 50 [1]. RA is often induced by an external agent (like cigarette smoking, infection or trauma) that triggers an autoimmune reaction, leading to synovial hypertrophy and chronic joint inflammation along with potential for extra-articular manifestations [1,2]. Although there are several associated mechanisms that trigger RA inflammation, the exact pathway that leads to it was not yet been determined [1,2,3].

Currently, there is no cure for RA, but some treatments are able to improve symptoms and hinder disease progression, helping to maintain joint functionality for daily activity [2,3,4]. Regarding RA diagnosis, magnetic resonance imaging (MRI) has emerged as a promising diagnostic tool for early disease detection [1,2,5]. Additionally, superparamagnetic iron oxide nanoparticles (SPIONs) have been proven to be very effective and versatile as MRI contrast agents for a variety of different applications [6]. Methotrexate (MTX) is one of the dominant drugs used in RA management, a low-cost disease-modifying anti-rheumatic drug that seems to exert its anti-inflammatory effects by acting at different levels of the pathophysiological signaling cascade [2,7]. MTX has proven to be effective against RA; however the non-specific administration of MTX leads to accumulation and subsequent damage of healthy tissues [1,2,4].

With the emergence of nanomedicine, many drug delivery systems have been developed, whose main objectives are the reduction of systemic side-effects and the maintenance of appropriate drug concentration in the required place [8,9,10,11,12,13]. Nanoparticles (NPs) have the potential to carry therapeutic and/or imaging agents, for theranostic applications. In particular, solid lipid nanoparticles (SLNs), due to their biocompatibility and biodegradability composed by physiologic lipids with Generally Recognized As Safe (GRAS) status and with a long history of use in pharmaceutical industry.

The possibility of NP multi-functionalization also opened the path for novel and widespread medical applications based on nanomedicine strategies, such as conjugation to specific ligands that target a specific tissue or organ or facilitate cellular penetration. Further functionalization can also be used to avoid/bypass bio-barriers and macrophage uptake, by coating the NP with polyethylene glycol (PEG), for example [11,12,13,14,15,16,17,18,19,20,21].

In this work, an approach to attempt targeted therapy and simultaneous imaging is proposed. This study aims to develop a targeted theranostic system for intravenous administration, consisting in the encapsulation of MTX (RA therapeutic drug) and SPIONs (contrast agent for MRI) within SLNs. These SLNs are functionalized with a monoclonal antibody (mAb) against the macrophage specific cell surface receptor, CD64, overexpressed in RA.

## 2. Results and Discussion

During the NP synthesis four different formulations were prepared: (i) cetyl palmitate SLNs; (ii) MTX-loaded cetyl palmitate SLNs; (iii) SPIONs-loaded cetyl palmitate SLNs and (iv) MTX- and SPIONs-loaded cetyl palmitate SLNs. All of these four formulations were then surface functionalized with an anti-CD64 antibody. In order for the formulations to be effective in the treatment and diagnostics of RA it is of the up most importance to ensure that both the therapeutic and the imaging agents, MTX and SPIONs respectively, are properly integrated into the NP.

### 2.1. Nanoparticle Characterization

The properties and characteristics of the NPs influence the formulation stability and can also influence their future interactions with cells and tissues [22,23], so they were characterized in terms of NP size, polydispersion index (PdI), Zeta potential as well as MTX content and SPIONs co-localization. These characteristics are summarized in Table 1 for all NP formulations.

**Table 1:** Physicochemical properties of the NP formulations. Size, PdI and Zeta potential for all the formulations and MTX association for the MTX-loaded and MTX- and SPIONs-loaded SLN prior to NP functionalization and post-functionalization.

Formulation	Size (nm)	PdI	Zeta Potential (mV)	MTX Association (%)
SLN	168 ± 7	0.234 ± 0.023	−39 ± 4	-
SLN + MTX	172 ± 6	0.211 ± 0.024	−35 ± 2 ^a^	98 ± 9
SLN + SPIONs	196 + 13 ^a^	0.238 ± 0.025	−35 ± 2 ^a^	-
SLN + MTX + SPIONs	199 ± 13 ^a^	0.246 ± 0.020	−33 ± 3 ^a^	102 ± 5
SLN + Anti-CD64	172 ± 4	0.225 ± 0.021	−17 ± 3 ^c^	-
SLN + MTX + Anti-CD64	167 ± 2	0.200 ± 0.024	−17 ± 2 ^c^	98 ± 9
SLN + SPIONs + Anti-CD64	196 ± 3 ^b^	0.184 ± 0.024	−18 ± 2 ^c^	-
SLN + MTX + SPIONs + Anti-CD64	206 ± 3 ^b^	0.209 ± 0.022	−17 ± 3 ^c^	102 ± 5

Values shown in mean ± standard deviation (*n* ≥ 3); ^a^ significantly different (*p* ≤ 0.05), between non-conjugated formulations comparing to SLN; ^b^ significantly different (*p* ≤ 0.05), between conjugated formulations comparing to SLN + Anti-CD64; ^c^ significantly different (*p* ≤ 0.05), between after and before functionalization.

#### 2.1.1. Nanoparticle Size, Polydispersion Index and Zeta Potential

All the NP formulations presented diameters between 150 and 210 nm (Table 1). It has been widely documented that NPs for intravenous applications should present sizes in the range of 100 to 300 nm. It has also been verified that particles around 250 nm are more easily incorporated into macrophages, which are the primary target cells of this proposed approach [24,25], making the developed NPs suitable for the intended administration route.

Regarding the non-conjugated formulations, the encapsulation of MTX did not influence significantly the size of the NPs. However, a significant increase in size can be observed when SPIONs were encapsulated into the SLNs. These results suggest that the SPIONs could be altering the structure and/or organization of the lipids in the SLN matrix. Conjugated formulations presented similar results to the non-conjugated NPs, as expected, considering that the antibody used is only around 10 nm, irrelevant to the mean NPs particle size obtained. Considering the size distribution profile, the PdI values, for all of the formulations considered, were below the reference value of 0.3 (Table 1), which is indicative of mono-dispersed NP populations presenting uniform NP diameters with little agglomeration [26].

Regarding the zeta potential results, all the prepared formulations presented distinct negative values (Table 1), expected when considering the lipids and surfactant used, cetyl palmitate, stearic acid and Tween 60. It has been described that negatively charged particles have reduced cellular uptake, due to electrostatic repulsion between them and the cellular membrane, however they show less cytotoxicity than cationic NPs that have been proven to be able to disrupt the cellular membrane and consequently lead to cell death [27].

An increase (value in modulus decrease) of the zeta potential after NP functionalization was confirmed. The antibody conjugation process was based on a reaction between the antibody’s primary amine and the fatty acids carboxylic group on the surface, providing two possible explanations for an increase in the zeta potential: (i) the antibody itself presents an overall positive charge that causes the particle’s charge to increase even if just slightly [28]; and (ii) the conjugation reaction causes a shielding around the carboxylic groups that are used to bind the antibody resulting in an increase of the zeta potential.

**Figure 1:**
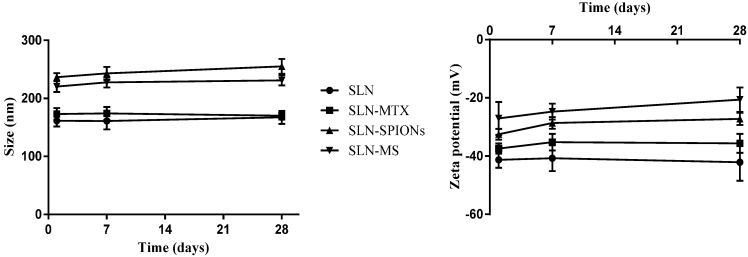
NP properties stability results showing NP size (**Left**) and Zeta potential (**Right**). Results shown as mean ± standard deviation (*n* = 18).

The stability of the NP formulations, regarding NP size and zeta potential, was also assessed over a period of twenty eight days and shown (Figure 1) that the formulations were able to maintain their properties over the studied time period. The results obtained were expected and suggest that the proposed formulations are viable and promising candidates for intravenous theranostic applications.

#### 2.1.2. Transmission Electron Microscopy

**Figure 2:**
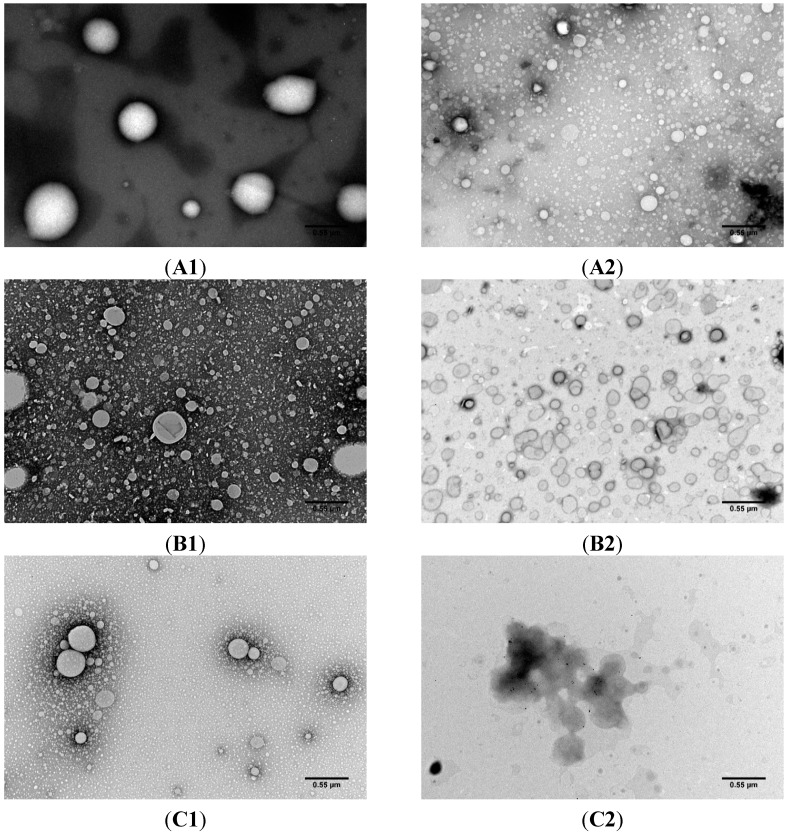

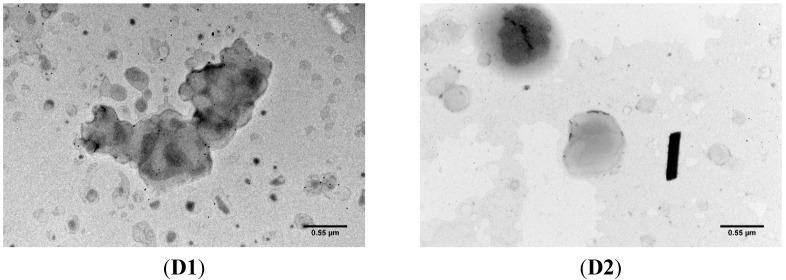
Transmission electron photographs of (**A1**,**A2**) cetyl palmitate NPs; (**B1**,**B2**) MTX-loaded cetyl palmitate NPs; (**C1**,**C2**) SPIONs-loaded cetyl palmitate and (**D1**,**D2**) MTX- and SPIONs-loaded cetyl palmitate. (**A1**,**B1**,**C1**,**D1**) non-conjugated NPs and (**A2**,**B2**,**C2**,**D2**) anti-CD64 conjugated NPs. Total magnification of 40,000× with a scale bar corresponding to 0.55 μm.

TEM photographs (Figure 2) were taken to corroborate NP size and assess NP morphology and SPION encapsulation. TEM allowed to confirm the sizes and PdIs that were previously measured using DLS when considering the placebo and MTX-loaded SLNs (Figure 2A1–B2). Additionally, it is possible to verify that MTX did not influence NPs morphology. The functionalization of the SLNs also didn’t seem to alter the shape of the SLNs that maintained their round spherical-shape (Figure 2A1–B2). The same could not be said for the formulations that were encapsulated with SPIONs (Figure 2C2–D2). These formulations showed disfigured NPs that in some cases formed agglomerates with undefined morphology, however these agglomerations observed help to explain the increase in size detected in the DLS results. A possible explanation for these disfigured and aggregated NPs could be connected to the fact that SPIONs were coated with oleic acid, leading to a lipid reorganization in the SLN matrix around the SPIONs. This re-organization could cause alterations to the SLN surface properties that resulted in the deformities and clustering of the SLNs.

The photographs taken by TEM (Figure 2C2–D2) were able to verify the encapsulation of the SPIONs inside the SLN matrix. The encapsulation of SPIONs within PLGA NPs has been described [8] and the results showed a considerable number of SPIONs within the NP matrix, primarily located in the core of the NPs. In this work solid lipids were used instead of PLGA and the results showed that NPs present fewer encapsulation of SPIONs and that they were found mainly in the NP periphery, indicating a different NP-SPION interaction.

#### 2.1.3. MTX Association Efficiency

The MTX association was determined by UV/Vis spectrophotometry, utilizing an indirect method, and rendered values higher than 98% for both MTX-loaded SLNs and for MTX- and SPIONs-loaded SLNS respectively. Both encapsulation values are similar and close to 100% encapsulation, indicating that the co-encapsulation of MTX and SPIONs is possible and may show promise for future theranostic applications.

#### 2.1.4. Antibody Conjugation

**Figure 3:**
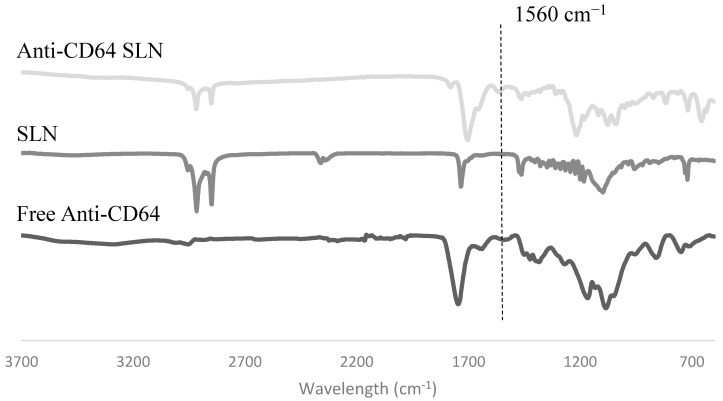
FT-IR spectrum of anti-CD64 conjugated SLNs, non-conjugated SLNs and free anti-CD64.

The FT-IR spectrum of anti-CD64-conjugated SLNs and SLNs were compared with the spectrum for anti-CD64 alone (Figure 3). FT-IR allows for the identification of atomic bonds by measuring their absorption—each bond has an absorption peak at a specific wavelength and these peaks are unique for each atomic bond [29]. On the anti-CD64 spectrum at 1560 cm^−1^ a peak can be observed, corresponding to a nitrogen-hydrogen bond [29]; this peak is not present on the formulations, as there are no amine groups in any of the other SLN components. By analyzing the spectrum of the anti-CD64 SLNs it is possible to observe a peak at around 1560 cm^−1^ indicating the presence of a nitrogen-hydrogen bond that, as expected, is not present in the non-conjugated SLNs denoting the presence of the antibody in the functionalized SLNs. A slight shift in the anti-CD64 conjugated-SLN and in the SLN can also be observed. In fact, the shifts FT-IR spectra usually occur due to alterations in the molecules or to the formation of complexes in the sample [30], suggesting that the developed SLN were successfully conjugated with the anti-CD64 antibody.

### 2.2. In Vitro Studies

In order to assess the effects of the developed NP formulations on cellular viability, MTT assay was performed on THP-1 cells. Regarding this assay the expected outcomes were that the SLN and the SPIONs-loaded SLN formulation would present no cytotoxicity since the components they contain have been shown to be biocompatible and the surfactant was not at high enough concentrations to be toxic [24,25,27,31]. Whereas the formulations that contained MTX (MTX-loaded SLNs and MTX- and SPIONs-loaded SLNs) should influence cellular viability and present cytotoxicity since MTX disrupts aspects of cellular metabolism and therefore decreases the cellular proliferation [7,8,32].

**Figure 4:**
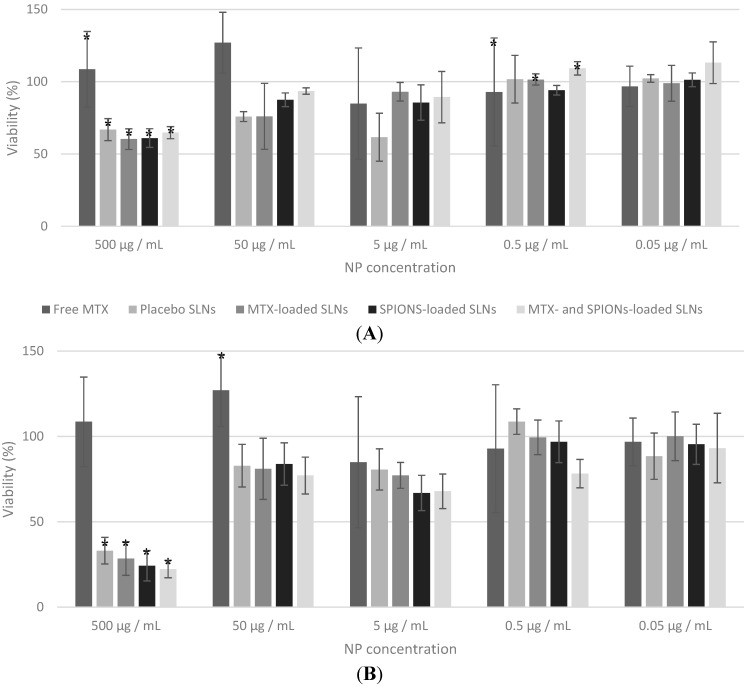
Effect of the developed NP formulations on differentiated THP-1 cells viability as a function of the different NP concentrations (500, 50, 5, 0.5 and 0.05 µg/mL) tested. Free MTX is diluted 100× relatively to the NP concentration to match MTX amounts present in the formulations. (**A**) Non-conjugated formulations and (**B**) conjugated formulations. Values represented as mean ± standard deviation (*n* ≥ 3; * statistical significant differences assessed with *p* ≤ 0.05).

The MTT assay (Figure 4) showed little variation between the cellular viability of cells incubated with NPs of different formulations (comparing among the same concentration). This could be explained considering the reduced amount of MTX added to the formulations that simply could have not been enough to achieve toxic levels. A similar effect, for same MTX concentration range, was previously observed [8]. MTX has been considered a cytotoxic agent that mediates its effect primarily on proliferating or cycling cells. However, it is pretty much accepted that in the present experiments, THP-1 cells are confluent, and when incubated with MTX or SLN the proliferation rate is low after 24 h. Thus, it was not expected high cytotoxicity as cells are not in intensive mitotic process.

On the other hand only the cells incubated with the highest concentrations of NPs impaired the cellular viability that confirms that the formulations are indeed biocompatible and well accepted by the cells. It is also important to note that the cells incubated with the higher concentrations of free-MTX showed greatly increased viability that was probably due to interferences cause by the large quantities of MTX present in the solution.

The antibody conjugated formulations (Figure 4B) presented no significant difference in viability when compared to non-conjugated ones (Figure 4A), except in the highest concentrations. These results show that the conjugation of SLNs with the anti-CD64 does not alter their biocompatibility.

The *in vitro* studies have proven that the proposed NP formulations do not impair cell viability and proliferation and present, therefore, potential and promise for biomedical applications, provided more extensive tests are performed.

## 3. Experimental Section

### 3.1. Materials

The lipids cetyl palmitate and Witepsol E85 were gently provided by Gattefossé (Saint Priest Cedex, France) and Sasol (Johannesburg, Gauteng, South Africa), respectively. Stearic acid was purchased from Merck KGaA (Darmstadt, Germany). MTX was obtained by courtesy of Excella GmbH (Feucht, Germany). Iron Oxide Nanocrystals (10 nm) coated with oleic acid and dispersed in chloroform (25 mg/mL) were kindly provided by Ocean Nanotech Inc. (Springdale, AR, USA). Anti-Human CD64 (Fc gamma Receptor 1) antibody solution (1 mg/mL) was purchased from eBioscience Inc. (San Diego, CA, USA). Tween 60, dimethyl sulfoxide ACS reagent ≥99.9% (DMSO), dichloromethane (ACS reagent, ≥99.5% contains 50 ppm amylene as stabilizer), ethyl acetate (ACS reagent, ≥99.5%), 2-morpholino-ethanesulfonic acid low moisture content, ≥99% (MES), 1-ethyl-3-(3-dimethylaminopropyl) carbodiimide hydrochloride purum ≥98.0% (EDC), N-hydroxysulfosuccinimide 98% (NHS), Thiazolyl Blue Tetrazolium Bromide 98% (MTT), Triton™ X-100 for molecular biology, Trypan Blue powder and phorbol 12-myristate 13-acetate (PMA) were purchased from Sigma-Aldrich (St. Louis, MO, USA). Dulbecco’s Modified Eagle’s Medium (DMEM), Dulbecco’s phosphate buffer saline 10× pH 7.4 (PBS), fetal bovine serum (FBS), Penicillin-Streptomycinm and Fungizone^®^ antimycotic were purchased from Gibco^®^ (Invitrogen Corporation, Paisley, UK). Aqueous solutions were prepared with double-deionized water (Arium Pro, Sartorius AG, Göttingen, Germany). THP-1 (ATCC^®^ TIB-202TM) cells were generously provided by Professor Susana Santos and Doctor Marta Freitas, Instituto Nacional de Engenharia Biomédica (INEB).

### 3.2. Methods

#### 3.2.1. Preparation of Multifunctional Nanoparticles

##### Nanoparticle Production Method

**Figure 5:**
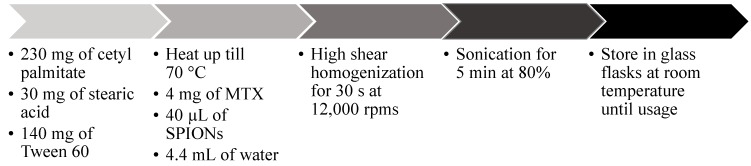
Schematic representation of the NP preparation procedure.

SLNs composed of cetyl palmitate and stearic acid were prepared by an organic solvent-free emulsification-sonication method that combined high shear homogenization and ultra-sonication (Figure 5). In more detail, the SLNs were composed of 57.5% cetyl palmitate, 7.5% stearic acid and 35% Tween 60. Firstly an extensive optimization process of the operating conditions for all the steps of the production method as performed (data not shown). As standard procedure, 230 mg of cetyl palmitate, 30 mg of stearic acid and 140 mg of Tween 60 were heated up to 70 °C (temperature above the fusion point of all the lipids in the mixture), in order to fuse the lipids. 4.4 mL of water at 70 °C were added to the fused lipid-surfactant mixture and the resulting solution was immediately homogenized with a High-shear Homogenizer (YSTRAL GMBH X10/20-E3, Ballrechten-Dottingen, Germany) for 30 s at 12,000 rpm to produce a lipid-in-water micro-emulsion. This micro-emulsion was afterwards re-homogenized using a probe-type sonicator (model VCX-130 with a VC 18 probe, Sonics & Materials Inc., Newtown, CT, USA) for 5 min at 80% amplitude to produce the NPs. The resulting NP suspension was stored in glass flasks at room temperature until further use.

The encapsulation of both MTX and SPIONs was achieved during the production method, both at 1% considering total SLN composition. 4 mg of MTX were added prior to the lipid fusing and dissolved, as much as possible, in fused solid lipid + surfactant mixture. 40 μL of SPION dispersion were added after the lipid fusion and before the addition of the water.

##### Antibody Conjugation to the Solid Lipid Nanoparticles

As previously stated, the SLN were further functionalized with an anti-CD64 antibody that is able to recognize macrophage’s CD64 (present in the macrophage cell membrane and over expressed in RA). This recognition is performed by the antibody’s Fc region and because of this any attempt of antibody conjugation must be done through the antibody’s Fab fragment in order to maintain its functionality. Considering this, the conjugation reaction performed in two steps: (i) the NP’s carboxylic groups (present in the stearic acid that was integrated into the SLN matrix) were activated in the presence of EDC and NHS; and (ii) the antibody’s primary amine (Fab fragment) reacted with the activated carboxylic group to form a stable amide bound between the SLN surface and the antibody [8].

The NP formulations were diluted in 10 mL of MES buffer, pH 5.0 (this value of pH ensures maximum EDC attachment to carboxylic groups), so that the final concentration is 10 mg of NPs per mL of buffer. The NPs were then activated by adding 1 mL of 0.1 M EDC and 1 mL of 0.7 M NHS (both dissolved in MES buffer, pH 5.0) to the previously prepared suspension. The NP suspension was then kept at room temperature under moderate stirring for 1 h. 1 mL of the previously prepared activated-NP suspension was transferred to an Eppendorf^®^ tube and 10 μL of the anti-CD64 antibody solution (1 mg/mL) were added to the latter. The resulting suspensions were then homogenized with a vortex and incubated at 4 °C for 24 h. The functionalized NPs were then stored at 4 °C until further usage.

#### 3.2.2. Nanoparticle Characterization

##### Dynamic Light Scattering and Phase Analysis Light Scattering

The NP formulations were characterized in terms of particle diameter, diameter distribution profile (PdI) and zeta potential. Mean diameter and PdI were determined by dynamic light scattering (DLS) using a 90Plus Particle Size Analyzer (Brookhaven Instruments Corporation, Holtsville, NY, USA) and the zeta potential was assessed by phase analysis light scattering (PALS) using a ZetaPALS Zeta Potential Analyzer (Brookhaven Instruments Corporation), at 660 nm, at 20 °C with a detection angle of 90°. The formulations were diluted in water, and all measurements were performed with three independent batches of NPs, with six runs each.

##### Transmission Electron Microscopy

Prior to the Transmission Electron Microscopy (TEM) analysis, the non-conjugated NP suspensions were diluted 100× in water to achieve a 1 mg/mL concentration, since the conjugated NPs were already at this concentration. Samples were prepared by placing 10 μL of NP dispersion on a copper-mesh grid and after 2 min the excess was removed with filter paper. As a contrasting agent, 10 μL of 0.75% uranyl acetate solution were placed on the grid and after 30 s the excess was removed using filter paper. The grids were after observed in a JEM-1400 Transmission Electron Microscope (JEOL Ltd., Tokyo, Japan), with an accelerating voltage of 80 kV.

##### MTX Quantification

UV/Vis spectrophotometry was used to determine the amount of MTX present in the formulations using an indirect method; *i.e.*, the amount of drug that was present outside the NPs was dosed, and the association efficiency was determined by subtracting the amount of MTX that remained in the aqueous phase to total amount of MTX added in the preparation of SLNs (Equation (1)):

(1) $$\text{Association efficiency }\left(\text{\%}\right)=\;\frac{\text{Total amount of MTX}-\text{Amount of MTX in the supernatant}}{\text{Total amount of MTX}}\times 100$$

Firstly the NP suspensions were diluted 200× and filtrated with Amicon^®^ Ultra Centrifugal Filters Ultracell-50 kDa (EMD Millipore, Darmstadt, Germany) at 3500 g for 30 min using a Heraeus™ Multifuge™ X1R centrifuge (Thermo Scientific, Waltham, MA, USA). The resulting supernatant was collected for MTX quantification by spectrophotometry. The absorbance of the supernatant was then measured using a V-660 UV/Vis Spectrophotometer (Jasco Inc., Easton, MD, USA), and the values of the absorbance were registered at 299 nm wavelength (MTX absorbance peak). The measurements of each sample, containing MTX, were corrected using the supernatant resulting from filtration of the respective formulation without MTX, in order to minimize formulation interferences. Each sample reading was performed in triplicate (each from an independent batch of NP formulations).

A calibration curve was constructed using standard solutions of MTX with the final concentrations of 0.625, 1.25, 2.5, 5, 10, 20, 30 and 40 μg/mL. In order to minimize reading interferences these standard solutions were prepared in the supernatant resulting from the previously described filtration of SLN NP suspension.

##### Fourier Transform Infrared Spectroscopy

Prior to Fourier transform infrared (FT-IR) analysis the NP formulations were lyophilized. Firstly 1 mL of NP suspension was frozen at −80 °C for 30 min and after placed in a lyophilizer Bench Top Pro with Omnitronics (SP Scientific, Warminster, PA, USA) at −80 °C at 150 m Torr for 18 h. The resulting powders were then characterized by FT-IR analysis, using a Frontier FT-IR Spectrometer with Universal ATR Sampling Accessory (PerkinElmer, Waltham, MA, USA). Each of the infrared spectrum was collect with 32-scans with 4 cm^−1^ resolution in the mid-infrared region (3700 to 600 cm^−1^).

#### 3.2.3. *In Vitro* Studies

THP-1 (ATCC^®^ TIB-202TM) cell line, cultured in DMEM medium supplemented with 10% FBS and 1% Penicillin-Streptomycin, incubated at 37 °C with 5% CO_2_. Cells were subcultured, centrifuging at 300 g and re-suspending in fresh medium. Cells were counted using a Neubauer chamber, diluted in a Trypan Blue solution 0.4% (*w*/*v*) in PBS in order to distinguish viable cells from the non-viable ones, and cultured in a new flask with a final concentration of 2 × 10^5^ cells/mL.

##### MTT Assay

THP-1 cells were seeded onto 96-well tissue culture test plates (Orange Scientific Products, Belgium), at a cellular density of 60 × 10^3^ cells/mL, supplemented with 0.02 μL/mL and incubated for 24 h. The next day the medium supplemented with PMA was discarded and replaced with fresh medium and incubated for another 24 h. The medium was then removed and 200 μL (100 μL of medium + 100 µL of PBS) of NP suspension were added with the final concentrations of 0.05, 0.5, 5, 50 and 500 μg of NPs per mL. A positive control (100 μL of medium + 100 µL of PBS) and negative control (200 µL of Triton™ X-100 2% (*w*/*v*) in PBS) as well as a control of free MTX (considering the potentially encapsulated MTX, with an association efficiency of 100% [1 mg of MTX per 100 mg of formulation] since MTX is highly hydrophobic at the pH used [33]) were also included to normalize and better compare results.

After 24 h of incubation the medium was removed and discarded. Then, 200 μL of MTT (0.5 mg/mL in culture medium) were added to each well and incubated for 4 h protected from the light. After 4 h the plate was removed from the incubator, the MTT solution was removed and 200 μL of DMSO were added, to dissolve the formazan crystals. The absorbance was then measured by using a Synergy™ HT Multi-mode Microplate Reader (Biotek Instruments, Winooski, VT, USA) at 590 nm and 630 nm, the latter was used for background subtraction. Cell viability was then determined using Equation (2):

(2) $$\text{Viability }\left(\text{\%}\right)=\;\frac{\text{Experimental Value}-\text{Negative Control}}{\text{Positive Control}-\text{ Negative Control}}\times 100$$

#### 3.2.4. Statistical Analysis

Statistical analyses were performed using IBM^®^ SPSS^®^ Statistics (SPSS 22.0, Armonk, NY, USA). Results are presented as mean value ± standard deviation from a minimum of three independent experiments. Two-tailed student’s *t*-test and one-way analysis of variance (ANOVA) were performed to compare dependent samples and multiple groups of independent samples, respectively. When the groups showed significant statistical difference (*p* ≤ 0.05), the differences between the respective groups were compared with a post-hoc test (Tukey, *p* ≤ 0.05). Paired (non-independent) samples were analyzed with a paired-samples two-tailed student’s *t*-test. Differences were once again considered significant at *p* ≤ 0.05.

## 4. Conclusions

The use of SLN for simultaneous *in vivo* imaging and drug delivery is an interesting approach for safe theranostic applications considering their biocompatibility and biodegradability, especially with the new possibilities of multifunctionalization brought by nanomedicine. In this work, an innovative attempt to achieve a combined therapy and imaging of RA was proposed, by co-encapsulating MTX and SPIONs inside cetyl palmitate and stearic acid SLNs. These SLNs were further functionalized with anti-CD64, an antibody that specifically binds to a cell surface receptor that is over expressed in RA infected macrophages.

The results obtained during this study showed that the proposed NP formulations presented interesting and promising results for future theranostics of RA, regarding both physicochemical properties of the NPs as well interaction with cell line THP-1 differentiated cells.

Considering the results shown, it can be seen that the proposed NP formulations may present an improved delivery of theranostic agents relying on safe and cheap SLNs, whose production can be easily scaled-up and tailored to achieve specific functions, such as directed targeting. Therefore, the proposed formulations have the potential to allow a more directed treatment as well as diagnosis and follow-up of the disease in a simpler and safer way, when compared with the current treatment and management of RA.

In conclusion, the results demonstrated that the proposed formulations are very promising for the future of RA treatment and diagnostic/monitoring, by providing a safe and targeted delivery of therapeutic agents and simultaneously allowing *in vivo* imaging. Still, more comprehensive and detailed studies should be performed in order for approaches such as this one to leave the lab and move on to the clinic, but the pavement is being laid for such a time.
